# Footprint of the eastern euroasian past in Italian populations of *Cryptotaenia thomasii* (Ten.) DC

**DOI:** 10.1186/s40529-016-0155-5

**Published:** 2017-01-02

**Authors:** Anna Maria Palermo, Liliana Bernardo, Domenico Gargano, Giuseppe Pellegrino

**Affiliations:** 1 Department of Biology, Ecology and Earth Sciences DiBEST, University of Calabria, 87036 Rende, CS Italy; 2 Museum and Botanic Garden, University of Calabria, 87036 Rende, Italy

**Keywords:** Apiaceae, *Cryptotaenia thomasii*, *Cryptotaenia flahaultii*, Genetic structure, SNPs

## Abstract

**Background:**

The knowledge of the genetic architecture of closely related species and/or populations of a single species can be very useful to shed light on the processes that led to their current distributions. The present study provides a preliminary evaluation of the conservation status of the populations of *Cryptotaenia thomasii*, a very narrow endemic species occurring in southern Italy. Previous studies showed that *C. thomasii* was phylogenetically closely related to *C. flahaultii,* endemic species of Caucasus, and to evaluate its conservation status and the genetic variability of plant species the internal transcribed spacers (ITSs) of nuclear ribosomal DNA were sequenced and the SNPs were analyzed.

**Results:**

The restriction analysis of nrDNA with the restriction enzymes allowed to detect the presence of two single mutations (SNPs) among the sequences of two species. Our molecular analysis pointed out that *C. thomasii* and *C. flahaultii*, in spite of their geographical disjunction, show a sign of an ancient contact as an extreme case of geographical disjunction Italian-Caucasus.

**Conclusion:**

From the evolutionary history of the species and its distribution pattern one can reconstruct a possible scenario with some hypotheses that explain the hypothetical ancestral area where the two species were in contact. We speculate the two species may have originated from fragmentation of a common ancestor widespread in the Western Palearctic zone and have survived in two separated refuge areas limited by important mountain systems. This hypothesis is supported by our molecular analysis, in fact, the analysis of SNPs showed that some *C. thomasii* populations retain the signs of an ancient link with *C. flahaultii*.

## Background

The mountains of Mediterranean basin, which were subject in the past to refuge area isolation processes as well as multiple biogeographical influences, constitute major centers of plant endemism and speciation (Thompson 2005). The populations of rare and endemic plant species deserve attention, especially when they are small and isolated, and then exposed to environmental events, demographic and genetic consequences developmentally harmful (Leimu et al. 2006; Gargano et al. 2009). Indeed, small populations can undergo genetic drift and biparental inbreeding with consequent loss of genetic diversity and fitness (Keller and Waller 2002; Reed and Frankham 2003; Gargano et al. 2015). Since some studies have estimated the size that allow a population to preserve much of their reproductive success and genetic diversity (Reed 2005), strategies for conservation of endangered species should be based on maintaining or increasing the size of their populations. Low or absent levels of gene flow increase the genetic difference between populations and the divergence of lineages could be favored by local adaptation (Morjan and Rieseberg 2004; Pickup et al. 2012). For this reason, biologists involved in conservation biology have used combined ecological and genetic data in order to identify those populations that represent significant evolutionary units and therefore require a conservation priority (Crandall et al. 2000; Stinchcombe and Hoekstra 2008).

The knowledge of the genetic architecture of closely related species and/or populations of a single species can be very useful to shed light on the processes that led to their current distributions. The variability and genetic structure of natural populations of plant species have been extensively evaluated by means of molecular markers. Molecular data, integrated with data from morphological, demographic, biological and climate, have allowed to correlate the genetic variability of plant species to historical and/or biological causes, often providing interesting insights for the understanding of the distribution of species (Petit et al. 1998; Thompson 1999).

The present study aims to provide a preliminary evaluation of the conservation status of the populations of the rare endemic *Cryptotaenia thomasii* (Ten.) DC. based on genetic data.

*Cryptotaenia thomasii* belongs to the Apiaceae, a family of dicotyledonous plants including 3700 species divided into 434 genera found in all temperate zones of the world. It is a family relatively homogeneous, characterized by a typical inflorescence: a simple or compound umbel. *Cryptotaenia* sensu lato is a small polyphyletic genus belonging to the subfamily Apioideae, tribe Oenantheae. It consists of eight species exhibiting a highly scattered distribution across continents (Spalik and Downie 2007). *Cryptotaenia africana*, *C. japonica* and *C. canadensis*, are widespread and occur in central and western Africa, eastern Asia, and eastern North America, respectively. The remaining species are narrow endemics and two of these species have Western Eurasian distribution: *C. thomasii* is a very narrow endemic species occurring in Calabria and Basilicata regions, southern Italy (Pignatti 1982); while *C. flahaultii* grows in the Caucasus (Tamamschian 1967). Both species were originally described in *Lereschia* (Boissier 1844) and then attributed to *Cryptotaenia* (Tutin 1968).

*Cryptotaenia flahaultii* has a very restricted range, grows on moist rocks in shaded forests up to 800 m asl. in only three populations located in Georgia, close the border with Turkey (Davis et al. 1972; Ketskhoveli et al. 1984). The total area of occupancy of this species is estimated to be approximately 12 km^2^, and the taxon is listed as Vulnerable under the D2 IUCN criterion (IUCN, 2012; Gagnidze 2014).

Mainly, *C. thomasii* occurs along mountain streams in forest areas, but in some cases it can colonize nitrogen-rich muddy habitats within mountain forests. The number of populations is low (ca. 20) and most of them account for a very low amount of individuals. The species is threatened by intrinsic factors, such as its restricted range and limited dispersal, by natural disasters such as drought, and by man-related pressures like forest exploitation, trampling and grazing. Based on the IUCN protocol of risk assessment (IUCN, 2012) it is precautionary assessed as Near Threatened (close to meet B2 requirements to fall into higher risk categories) (Ali 2010). Genetic data (Pellegrino et al. unpublished) showed that *C. thomasii* and *C. flahaultii* were phylogenetically closed related species.

This endangered species occurs in a few small populations, which are exposed to multiple pressures that threaten the species’ persistence. Genetic data for *C. thomasii* will allow informed conservation decisions involving satellite population establishment, population augmentation, and prioritization of conservation efforts. It was therefore conducted a molecular analysis on individuals belonging to 10 populations in order to assess levels and distribution of genetic variation within and between populations. For this purpose, were sequenced the Internal Transcribed Spacers (ITSs) of nuclear ribosomal DNA and analyzed the SNPs using appropriate restriction endonucleases.

## Methods

**Fig. 1:**
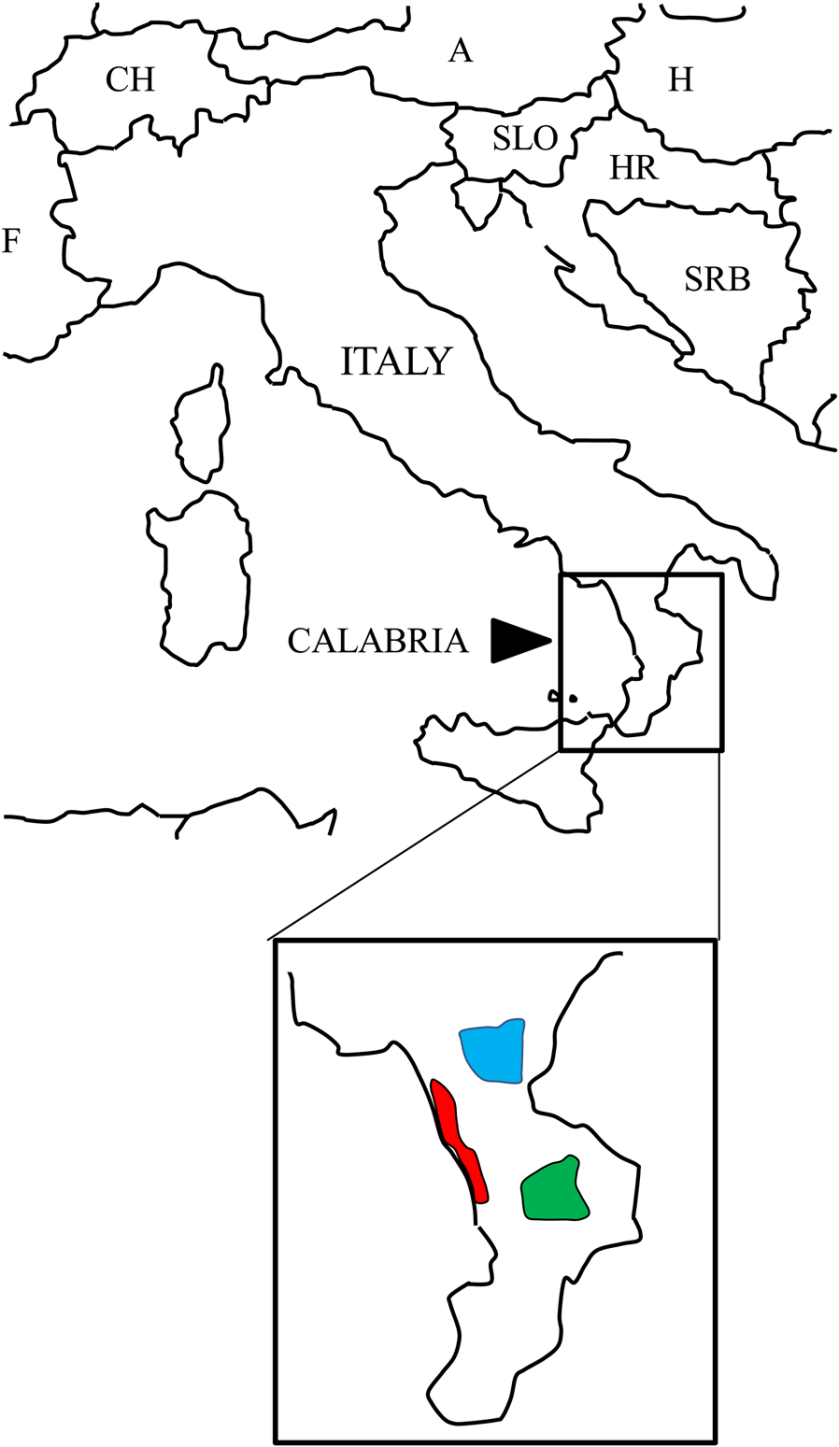
Geographic distribution of Italian populations of *Cryptotaenia thomasii*, sampled in three region (Sila *blue*, Catena Costiera *red* and Pollino *green*)

To perform molecular analysis, leaf tissue samples of *C. flahaultii* was obtained from individuals of the three populations in Georgia, while leaf tissue samples of *C. thomasii* were collected in spring of 2013 from individuals of ten populations (Table 1). Voucher specimens were deposited at the herbarium at the University of Calabria (CLU). The ten populations were split into three regions based on geographic proximity (Sila, Catena Costiera and Pollino) (Fig. 1). Sample sizes were based on collection permits and varied depending on the population size. Overall, leaves from 10 to 20 individuals from each population were carefully removed, immediately placed into collection tubes with silica gel, and stored at room temperature.

**Table 1:** Characteristics of the studied populations of *Cryptotaenia thomasii*

	Populations	Region	Latitude	Longitude	Altitude (m)
1	Cannavino	Sila	39°.18′N	16°.20′E	750
2	Celico		39°.18′N	16°.19′E	680
3	Mt Scuro		39°.19′N	16°.23′E	1100
4	Cecita		39°.21′N	16°.30′E	1000
5	Falconara	Catena Costiera	39°.16′N	16°.06′E	800
6	Silo		39°.14′N	16°.09′E	700
7	Laghicello Fuscaldo		39°.24′N	16°.04′E	1000
8	Mt Caramola	Pollino	39°.55′N	16°.12′E	900
9	San Severino		40°.01′N	16°.19′E	1200
10	Mancini		40°.00′N	16°.11′E	1100

Total genomic DNA was isolated from dried leaf tissue using the cetyltrimethyl ammonium bromide (CTAB) method (Doyle and Doyle 1987). Approximately 50 mg of each leaf were separately pestled in a 2 mL-eppendorf vial using 500 μL of CTAB buffer, incubated at 60 °C for 30 min, extracted twice adding 500 μL of chloroform-isoamyl alcohol (24:1), precipitated with 350 μL of isopropanol and washed with 150 μL of ethanol 70%. After extraction, the DNA was resuspended in 50 μL of 1× Tris–EDTA (TE) buffer, and the concentration was estimated by comparison to a known standard on a 0.8% agarose gel. DNA extractions were stored at 4 °C short term and at −80 °C for long-term storage.

The nuclear ribosomal internal transcribed spacers (ITS1 and ITS2+) were amplified by polymerase chain reaction (PCR) using universal pairs of primers as described in Pellegrino et al. (2001). PCRs were carried out in a total reaction volume of 100 μL, containing approx. 10–20 ng of DNA, 100 μL of reaction buffer 1× , 2 mM MgCl_2_, 100 mM of each dNTP, and 2.5 Units of BioTaqTM DNA Polymerase (Bioline Inc. Boston, MA, USA), and 0.2 mM of each primer (MWG-Biotech AG, Ebersberg, Germany). The thermocycling profile consisted of an initial denaturation step at 94 °C for 3 min, followed by 30 cycles with 30 s at 94 °C, 30 s at 55 °C, and 2 min at 72 °C. PCRs were performed on a PTC-100 Thermal Cycler (MJ Research Inc. Watertown, MA, USA). PCR fragments were purified by QIAquick PCR purification kit (Qiagen S.p.A. Milan, Italy) to remove unincorporated primers and dNTPs. Amplification products were electrophoretically separated on a 2% agarose gel, compared to a 100 base pair (bp) ladder (Pharmacia Biotech) as the molecular weight marker, stained with ethidium bromide and photographed using a Kodak digital camera. Nuclear amplified fragments of three individuals for each population were sequenced in both directions using a modification of the Sanger dideoxy method as implemented in a double stranded DNA cycle sequencing system with fluorescent dyes. Sequence reactions were then run on a 373 A Applied Biosystems Automated DNA sequencer (Applied Biosystems, Foster City, CA, USA). Nuclear sequences were examined using GeneJockey to find a restriction site that would distinguish them using Polymerase Chain Reaction-Restriction Fragment Length Polymorphism (PCR–RFLP). This approach allows the examination of a single nucleotide polymorphism (SNP) without the necessity of cloning and subsequently sequencing several ITS clones.

Restriction enzymes *Mbo*I e *Apa*I, which cut at 5′-GATC/-3′ and 5′-GG/CC-3′, differentiated the sequences due to the presence of a C/T substitution about 60 base pairs into the ITS2 sequence, and to the presence of a C/A substitution about 78 base pairs into the ITS1 sequence, respectively. Thus, the PCR fragments of all samples (100 ng) were digested in a final 20 μL volume with the selected restriction endonuclease (1 U/ng DNA), according to the manufacturer’s instructions (Fermentas), incubated for 3 h at 37 °C. The fragments were electrophoretically separated on a 3% low melting agarose gel (Methaphore, FMS), compared to a 100 base pair (bp) ladder (Pharmacia Biotech) as the molecular weight marker, stained with ethidium bromide and photographed using a Kodak digital camera.

## Results

Overall the length of the sequence of the ribosomal DNA of *C. thomasii* and *C. flahaultii* is 598 base pairs in length, in particular the ITS1 is 210 bp, the 5.8 S 163 bp and ITS2 225 bp long. The G + C content of ITSs was found to be 57.14% (ITS1) and 59.11% (ITS2). Sequences of both species were deposited in the GenBank (ID 1953447 and 1953454).

The restriction analysis of nrDNA with the enzymes *Mbo*I and *Apa*I allowed to detect the presence of two single mutations (SNPs) among the sequences of *C. thomasii* and *C. flahaultii*.

The electrophoresis showed a different pattern due to the presence or absence of cleavage sites of the enzymes. Indeed, ITS2-containing fragments digested with *Mbo*I showed a single restriction site in all individuals of *C. flahaultii* and in few individuals belonging to two out of 10 populations of *C. thomasii* (populations Cannavino and Mt Scuro) belong belonging to Sila region and no site in other *C. thomasii* populations. Similar results have been highlighted using *Apa*I which showed a single restriction site in ITS1 of all individuals of *C. flahaultii* and in some individuals belong to two populations (Cannavino and Mt Scuro) of *C. thomasii* and no site in other *C. thomasii* populations. In details, three individuals of pop. Cannavino and four individuals of pop. Mt Scuro of *C. thomasii* exhibited a direct additive inheritance of these profiles, their digested fragments produced the combination of diagnostic profiles obtained for both species. These individuals showed a gel electrophoresis pattern with three band, one of the same length of full amplification product and two resulting from the digestion ITS amplicon. In addition, it is evident that the two SNPs of the two populations of *C. thomasii* are sites of addictivity (heterozygosity) nucleotide, showing the simultaneous presence of a base inherited from one species and the other from the second species. Indeed, in position 78 of ITS1 *C. thomasii* had a C and *C. flahaultii* an A while the sequences of few individuals of two populations of *C. thomasii* showed a M (C + A), and in position 60 of ITS2 *C. thomasii* showed a C and *C. flahaultii* a T while few individuals of two populations of *C. thomasii* a Y (C + T) (Table 2).

**Table 2:** SNPs in ITS 1 and ITS 2 sequences of *Cryptotaenia thomasii* and *C. flahaultii*

	ITS 1					ITS 2				
	76	77	78	79	80	58	59	60	61	62
*C. thomasii*	G	G	C	C	G	A	T	C	G	T
Heterozygosity			M					Y		
*C. flahaultii*	G	G	A	C	G	A	T	T	G	T

## Discussion

Our molecular analysis pointed out that *Cryptotaenia thomasii* and *C. flahaultii*, in spite of their geographical disjunction, show a sign of an ancient contact.

The genus *Cryptotaenia* is characterized by a particular pattern of geographical distribution (Spalik and Downie 2007), with three species (*C. africana*, *C. japonica* and *C. canadensis*) widely distributed, while the remaining five are endemic geographically isolated from their putative parents. Among the latter, two in particular have a disjunct distribution in the Western Palearctic, *C. thomasii* is endemic to southern Italy (Pignatti 1982) and *C. flahaultii* instead the Caucasus (Tamamschian 1967). Such regions were main areas of refuge for the Tertiary flora (about 50 million years ago) in Europe (Willis 1996; Fauquette et al. 1999) and Asia (Grossheim 1948; Milne and Abbott 2002). Other herbaceous species are known with a disjunct distribution similar such *Calamintha grandiflora* Moench (Lamiaceae), *Digitalis ferruginea* L. (Plantaginaceae) and *Salvia glutens* L. (Lamiaceae), which spread from Italy and neighboring France up to the Carpathian passing from the Balkans and the Black Sea coast (Meusel et al. 1978; Tzonev et al. 2005), but that of *C. thomasii* and *C. flahaultii* is really an extreme case of geographical disjunction Italian-Caucasus.

It is interesting to discuss the biogeographic point of view due to the geographical distance that currently exists between the populations of *C. thomasii* and *C. flahaultii*.

The particular geographical pattern shown by *Cryptotaenia.* and many other genera of temperate regions represents the distribution of relict Tertiary (Cenozoic), the result of complex and often intertwined processes that occurred in the past, such as migration, dispersal, vicariance, speciation and extinction (Xiang et al. 1998; Wen 1999, 2001; Donoghue et al. 2001; Xiang and Soltis 2001).

In an attempt to reconstruct a possible scenario that can explain the hypothetical ancestral area where the two species were in contact and the current disjunct distribution can make some hypotheses.

A plausible scenario is that *Cryptotaenia s.s.* was originated in East Asia and later there were two events of dispersion, the first westward to Europe, the second to the North America (Spalik and Downie 2007). When moving to Europe so it would be to spot the large morphological and genetic identity between *C. thomasii* and *C. flahaultii*. But this hypothesis provides a great ability of dispersion. Conversely species in the group have a poor ability to seed dispersal. In fact, their fruits are hairless and show no structure to improve the dispersion which is then entrusted only to gravity and wind. It seems so unlikely that there may have been a long-distance dispersal since the fruits/seeds are no carried by animals. Indeed dispersions in a great distance have been demonstrated for species whose seeds are dispersed by zoochory (Cain et al. 2000; He et al. 2010).

Alternatively, the two species may have originated from fragmentation of a common ancestor widespread in the Western Palearctic zone, including Europe, northern Africa and western Asia. In this way, during the ice ages from Pliocene (about 2 million years ago), *C. thomasii* and *C. flahaultii* would have survived in two separated refuge areas, by giving a little contribution to subsequent post-glacial colonization. For instance, *C. thomasii*, confined in a small refuge area of southern Italy, did not contribute to the post-glacial colonization of Europe since the Alps are an important barrier to migration. For example, species such as the oaks were able to colonize Europe coming from refuges of the Iberian Peninsula or the Balkans, with a limited contribution from the Italian refuges (Petit et al. 2002). Probably, the aforementioned poor dispersal ability along with a narrow ecological niche trapped *C. thomasii* in South Italy, by limiting chance to colonize neighboring areas. This hypothesis is supported by our molecular analysis. In fact, the analysis of SNPs showed that some *C. thomasii* populations retain the signs of an ancient link with *C. flahaultii* as would be expected in presence of a common ancestor. The sequences of individuals of two populations showed two sites of nucleotide additivity due to an ancient gene flow between the two species so that in the past were necessarily in contact. The molecular data is also in line with the morphological “*stasi*”, i.e. the great morphological similarity between *C. thomasii* and *C. flahaultii*. The low dispersion capacity and the ecological requirement limited the ability to conquer new areas and increase gene flow, and the consequent difficulty of overcoming geographical barriers (sea to the south, the mountains to the north) created a relict species in which some populations preserved in their the genotype traces of the past.

## Conclusion

Then, the limitations in reaching new sites due to dispersal and adaptative constrains played a major role in the evolutionary history of *C. thomasii*, by shaping its current patterns of distribution and phenotypic relationship with the closest relative. However, the same limitations have strong implication for the effective conservation of such narrow endemic. In presence of habitat perturbation plant may rely on two main strategies: colonization of new sites characterized by more favourable conditions, or a adaptations to the novel ecological context. Nonetheless, species showing natural-history traits similar to *C. thomasii* (low dispersal ability, strong ecological specialization) are likely to withstand adverse habitat conditions by both the mentioned ways.
